# HIF2α regulates the synthesis and release of epinephrine in the adrenal medulla

**DOI:** 10.1007/s00109-021-02121-y

**Published:** 2021-09-04

**Authors:** Deepika Watts, Nicole Bechmann, Ana Meneses, Ioanna K. Poutakidou, Denise Kaden, Catleen Conrad, Anja Krüger, Johanna Stein, Ali El-Armouche, Triantafyllos Chavakis, Graeme Eisenhofer, Mirko Peitzsch, Ben Wielockx

**Affiliations:** 1 Institute of Clinical Chemistry and Laboratory Medicine, Technische Universität Dresden, Fetscherstrasse 74, 01307 Dresden, Germany; 2 Department of Experimental Diabetology, German Institute of Human Nutrition Potsdam-Rehbruecke, 14558 Nuthetal, Germany; 3 German Center for Diabetes Research (DZD), 85764 München-Neuherberg, Germany; 4 Department of Pharmacology and Toxicology, Medical Faculty, Technische Universität Dresden, 01307 Dresden, Germany; 5 Department of Medicine III, Medical Faculty, Technische Universität Dresden, 01307 Dresden, Germany

**Keywords:** Hypoxia inducible factor, Catecholamines, Oxygen sensors, Glucose

## Abstract

**Abstract:**

The adrenal gland and its hormones regulate numerous fundamental biological processes; however, the impact of hypoxia signaling on adrenal function remains poorly understood. Here, we reveal that deficiency of HIF (hypoxia inducible factors) prolyl hydroxylase domain protein-2 (PHD2) in the adrenal medulla of mice results in HIF2α-mediated reduction in phenylethanolamine N-methyltransferase (PNMT) expression, and consequent reduction in epinephrine synthesis. Simultaneous loss of PHD2 in renal erythropoietin (EPO)-producing cells (REPCs) stimulated HIF2α-driven EPO overproduction, excessive RBC formation (erythrocytosis), and systemic hypoglycemia, which is necessary and sufficient to enhance exocytosis of epinephrine from the adrenal medulla. Based on these results, we propose that the PHD2-HIF2α axis in the adrenal medulla regulates the synthesis of epinephrine, whereas in REPCs, it indirectly induces the release of this hormone. Our findings are also highly relevant to the testing of small molecule PHD inhibitors in phase III clinical trials for patients with renal anemia.

**Key messages:**

- HIF2α and not HIF1α modulates PNMT during epinephrine synthesis in chromaffin cells.
- The PHD2-HIF2α-EPO axis induces erythrocytosis and hypoglycemia.
- Reduced systemic glucose facilitates exocytosis of epinephrine from adrenal gland.

**Supplementary Information:**

The online version contains supplementary material available at 10.1007/s00109-021-02121-y.

## Introduction

The hypoxia signaling pathway regulates expression of myriad genes involved in various biological processes in living animals; the hypoxia inducible factors (mainly HIF1 and HIF2) are the central transcription factors that regulate these processes. HIF expression, in turn, is under the direct control of a set of oxygen sensors known as the HIF-prolyl hydroxylase domain-containing proteins (PHD1-3). Under normoxic conditions, PHDs use oxygen as a co-factor to hydroxylate two prolyl residues in the HIFα subunits, thereby making HIFs accessible to the von Hippel-Lindau protein complex (pVHL) for subsequent ubiquitination and degradation [1]. Reduced cellular oxygen levels preclude such hydroxylation of HIFs by PHDs, resulting in stabilization of HIFα and direct transcriptional activation of more than 1000 genes. HIF transcriptional targets are primarily involved in a wide range of biological processes that serve to reverse the unfavorable hypoxic state, including erythropoiesis, blood pressure regulation, and adrenocortical hormone production [2–4]. Hypoxia is also a central feature of multiple pathologies, including local and systemic inflammation, and various stages of tumorigenesis or metastatic progression; gene mutations that impact hypoxia signaling are particularly important for development of pheochromocytomas, tumors originating from the adrenal chromaffin cells [5].

Adrenal chromaffin cells are the principal endocrine cells of the adrenal medulla and, as the source of catecholamines, are crucially involved in the fight-or-flight response, which requires epinephrine secretion [6]. Biosynthesis of epinephrine is catalyzed by the enzyme phenylethanolamine N-methyltransferase (PNMT), which converts norepinephrine to epinephrine. Several in vitro studies have focused on HIF involvement in regulating the enzymatic activity required for catecholamine synthesis, including a role for HIF2α in the expression of dopamine β-hydroxylase (DBH) and Dopa decarboxylase (DDC) in immortalized rat fetal adrenal medullary cell lines [7]. Conversely, another in vitro study found no impact of HIF2α expression on tyrosine hydroxylase (TH) or Dbh [8], while downregulating PNMT expression [9]. The latter finding is in line with results from a number of other studies that have connected HIF2α activity in the adrenal medulla or in pheochromocytomas with reduced PNMT production [9–11]. Tumors with pVHL mutations also overexpress HIF2 target genes such as erythropoietin (EPO), a hormone central to red blood cell (RBC) formation [12, 13]. Despite these observations, studies focusing on how modulation of hypoxia pathway proteins affect adrenal function are sparse, and only recently has a transgenic mouse line harboring a whole body HIF2α gain-of-function mutation been described, which showed reduced PNMT in the adrenal glands [14].

Circulating concentrations of epinephrine during hypoglycemia can increase up to 30-fold. Actually, hypoglycemia stimulates the adrenal medulla rather specifically compared to the neuronal components of the sympathetic nervous system [15–17]. Hyperglycemia is associated with inhibition of insulin exocytosis from β-cells [18, 19] but has also been suggested to inhibit the release of epinephrine [20]. Interestingly, elevated levels of systemic EPO or treatment with erythropoiesis stimulating agents are directly linked to a reduction in blood glucose levels (BGLs), possibly due to higher glucose consumption by increased numbers of RBCs [21–23]. As the production and release of epinephrine are critical, in vivo studies are essential to better understand the direct and/or indirect impact of alterations in hypoxia pathway proteins on epinephrine production and release from the adrenal gland.

Here, we performed an in-depth study of the synthesis of epinephrine and its release from the adrenal gland using several transgenic mice models that exhibit functional changes in one or more hypoxia pathway proteins. Our results demonstrate that PHD2 deficiency in the adrenal medulla, using our previously described CD68:cre-PHD2^f/f^ and PHD2/HIF1α^ff/ff^ mouse lines [24, 25], results in HIF2α-mediated reduction in PNMT and consequent inhibition of epinephrine synthesis in the adrenal gland. Moreover, we show that differential secretion of epinephrine from the adrenal gland is dependent on EPO/RBC-induced changes in blood glucose levels.

### Materials and methods

#### Mice

All mouse strains were maintained under specific pathogen-free conditions at the Experimental Centre of the Medical Theoretical Center (MTZ, Technical University of Dresden—University Hospital Carl-Gustav Carus) or at the animal facility of Max Planck Institute of Molecular Cell Biology and Genetics (MPI-CBG), Dresden. Experiments were performed with both male and female mice aged between 6 and 12 weeks. No significant differences between the genders were observed. CD68:cre-PHD2/HIF1^ff/ff^ (P2H1) or CD68:cre-PHD2^f/f^ (P2) lines were generated in-house and have been previously described by us. Briefly, although CD68 is known as a monocyte/macrophage marker, cre-recombinase activity/targeting was found in the entire hematopoietic system (starting in the hematopoietic stem cell), a few subsets of epithelial cells, neurons, and EPO-producing cells in the kidney (REPC) [24, 25]. CD68:cre-HIF1^f/f^ mice were generated for the current work using the HIF1^f/f^ line [26]. EPO Tg6 mice have been described earlier [27]. FOXD1:cre-HIF2α^f/f^ mice have been described elsewhere [28] but were generated in our laboratory using the FOXD1:cre line [29] (generous gift from Dr. Todorov, Dresden, Germany) in combination with the HIF2^f/f^ line [30]. All mice described in this report were born in normal Mendelian ratios. Mice were genotyped using primers described in supplementary Table 1. Peripheral blood was drawn from mice by retro-orbital sinus puncture using heparinized microhematocrit capillaries (VWR, Germany), and plasma separated and stored at −80 °C until further analysis. Urine was immediately frozen on dry ice after collection and stored at −80 °C until further analysis. Mice were sacrificed by cervical dislocation and adrenals were isolated, snap frozen in liquid nitrogen, and stored at −80 °C for hormone analysis or gene expression analysis. All mice were bred and maintained in accordance with facility guidelines on animal welfare and with protocols approved by the Landesdirektion Sachsen, Germany.

#### Laser microdissection

To investigate genetic targeting of hypoxia pathway proteins in the CD68:cre line, we isolated the medullar fraction of the adrenal gland using laser microdissection. Therefore, adrenal glands were embedded in O.C.T Tissue-Tek (A. Hartenstein GmbH) and subsequently frozen on dry ice and stored at −80 °C until further processing. Serial sections of 25–30 µm were obtained at −14 °C using a cryotome. The glass slides used for collection of sections were heated at 200 °C for a minimum of 3 h and UV treated for 15 min. After collection of sections, slides were subjected to sequential dehydration in H_2_O, 75% EtOH, 95% EtOH, and 100% EtOH for 1 min each and then left to dry completely. Microdissection of the adrenal medulla was performed using a PALM MicroBeam LCM (ZEISS). Tubes containing the tissue were frozen to −20 °C until further processing and genomic DNA was isolated from the dissected tissue using the alkaline lysis buffer followed by the neutralization buffer and used as template for genomic PCR.

#### Hormone measurement

Adrenal glands were incubated in disruption buffer (component of Invitrogen™ Paris™ Kit, AM 1921, Thermo Fisher Scientific) for 15 min at 4 °C, homogenized in a tissue grinder, followed by incubation for 15 min on ice and further preparation [31]. Adrenal *catecholamines*, norepinephrine, epinephrine, and dopamine were measured by high pressure liquid chromatography (HPLC) coupled with electrochemical detection after batch extraction with alumina, as previously described [32]. *Urinary catecholamines* were determined by liquid chromatography tandem mass spectrometry (LC–MS/MS) as described previously [33]. All urine samples were normalized for their volume by urinary creatinine measurement.

#### PNMT enzyme activity

PNMT enzyme activity was analyzed by LC–MS/MS [34].

#### RNA extraction and qPCRs

RNA from the adrenal glands was isolated using the RNA Easy Plus micro kit (Qiagen). cDNA synthesis was performed using the iScript cDNA Synthesis Kit (BIO-RAD). Gene expression levels were determined by quantitative real-time PCR using the “Ssofast Evagreen Supermix” (BIO-RAD). Primer sequences used for qPCRs are included in supplementary Table 2. Expression levels of genes were determined using the Real-Time PCR Detection System-CFX384 (BIO-RAD). All mRNA expression levels were calculated relative to housekeeping genes, β2M or EF2, and were normalized using the ddCt method. Relative gene expression was calculated using the 2(-ddCt) method, where ddCT was calculated by subtracting the average WT dCT from dCT of individual samples.

#### Immunofluorescence analysis

For immunofluorescence staining, frozen sections of adrenal glands were stained with an Anti-Tyrosine Hydroxylase antibody (ab76442) from Abcam. For additional visualization of the nuclei, cells were covered with DAPI. Fluorescent images were acquired on an ApoTome II Colibri (Carl Zeiss, Jena, Germany). Images were analyzed using Fiji (ImageJ distribution 1.52 K) to quantify staining per region of interest (ROI).

#### Cell culture

Mouse pheochromocytoma cells (MPC — 4/30/PRR) were obtained from Arthur Tischler (Department of Pathology and Laboratory Medicine, Tufts University School of Medicine, Boston, MA, USA; and Dr. Pacak, NIH, Bethesda, MD) [35]. The MPC cells were plated on collagen A-coated flasks (Biochrom AG, Berlin, Germany) and maintained in RPMI 1640 medium containing 5% fetal calf serum and 10% horse serum (all from Life Technologies, Darmstadt, Germany) at 37 °C, 95% humidity, and 5% CO_2_.

#### Intracellular calcium uptake

MPCs were cultured in 24-well plates in low serum medium — Opti-MEM (Thermo Fisher Scientific). Cells were plated onto collagen-coated plates in Opti-MEM for 3 h and subjected to respective treatment conditions of either glucose (D-( +)-glucose) (Sigma Aldrich) or erythropoietin (Roche). Cells were harvested 24 h later, washed, transferred to a 96-well plate, and subjected to staining with 0.5 µmol Fluo-8-AM (Abcam) for 60 min at 37 °C in assay buffer (HBSS with Pluronic F127 plus), followed by washing with HBSS and analysis on a BD FACS Canto.

#### Statistical analyses

All data are presented as mean ± SEM. Data (WT control versus transgenic line) were analyzed using the Mann–Whitney *U*-test, or the unpaired *t*-test with Welch’s correction, as appropriate (after testing for normality with the *F* test). All statistical analyses were performed on GraphPad Prism ver. 7.02 for Windows (GraphPad Software, La Jolla, CA, USA, www.graphpad.com). Significance was set at *p* < 0.05; “*n*” in figure legends denotes individual samples.

### Results

#### Alterations in hypoxia pathway proteins reduce adrenal epinephrine

**Fig. 1:**
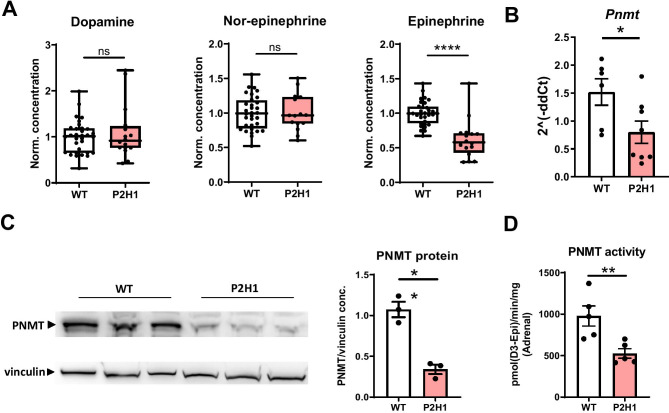
Loss of PHD2 and HIF1 results in decreased epinephrine synthesis and PNMT activity in the adrenal gland. **A** Box and whisker plots showing catecholamine measurements in the adrenals from WT mice in comparison to littermate P2H1 mice (*n* = 16–31 individual adrenal glands). All data is normalized to average measurements in WT mice. The graphs are a representative result of at least 3 independent experiments. **B** qPCR-based mRNA expression analysis of *Pnmt* in the adrenal gland from individual P2H1 mice and WT littermates (*n* = 6–8 individual adrenals). **C** Western blot analysis and comparison of PNMT protein from the adrenals of P2H1 mice and WT controls (*n* = 6 vs 3 individual adrenals). **D** PNMT enzyme activity in the adrenals of P2H1 mice compared to the WT littermate controls. Statistical significance was defined using the Mann–Whitney *U*-test (*p < 0.05; **p < 0.005)

Previously, we described a mouse line with conditional PHD2 and HIF1α inactivation in a variety of cells (CD68:cre-PHD2/HIF1α^ff/ff^—henceforth designated P2H1), including neurons and renal EPO-producing cells (REPC), which results in local HIF2α stabilization that leads to excessive systemic EPO and erythrocytosis (Supplementary Fig. 1A) [25]. As these cell lineages are thought to derive from neural crest cells [28, 36, 37], we looked at the effects of modulating hypoxia pathway proteins in another neural crest-derived cell type, namely, chromaffin cells, which are located in the medulla of the adrenal gland. First, to confirm genetic targeting of both hypoxia pathway proteins, we performed genomic PCRs on laser microdissected adrenal medullary tissue confirming localized targeting of PHD2 and HIF1α (Supplementary Fig. 1B). Furthermore, qPCR analysis using mRNA from whole adrenal glands showed reduction of *Phd2* and *Hif1α* compared to the WT littermate controls, and an associated increase in *Hif2α* (Supplementary Fig. 1C). Next, catecholamine levels in adrenal gland lysates showed a dramatic decrease in only epinephrine in P2H1 mice compared to their WT littermates, but not of the upstream hormones, namely, dopamine and norepinephrine (Fig. 1A). We also observed a corresponding marked decrease in *Pnmt* mRNA, protein, and enzymatic activity (Fig. 1B–D). Conversely, no differences in the expression of other catecholamine-associated enzymes, such as *Th* or *Dbh*, were detected (Supplementary Fig. 1D). In addition, we found no difference in the amount of TH-protein using immunofluorescence staining (Supplementary Fig. 1E). Taken together, these results show that inhibition of the oxygen sensor PHD2 and one of its downstream HIF targets in the adrenal gland leads to diminished epinephrine synthesis.

#### Decreased adrenal epinephrine synthesis is unrelated to loss of *HIF1a*

**Fig. 2:**
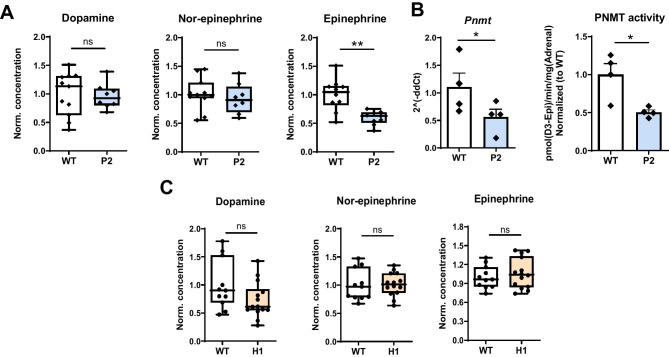
Decreased epinephrine and PNMT in the adrenal are related to the loss of PHD2 but unrelated to HIF1. **A** Box and whisker plots comparing catecholamine measurements in the adrenals from P2 mice and WT littermates (*n* = 8–11 individual adrenal glands). All data are normalized to average measurements in WT mice. The graphs are a representative result of at least 3 independent experiments. **B** qPCR-based mRNA expression analysis of *Pnmt* and PNMT activity in the adrenals of the individual mice (*n* = 4 vs 4). **C** Catecholamine measurements in the adrenals of HIF1 mice compared to WT controls (*n* = 11–14 individual adrenal glands). All data are normalized to the average of measurements in WT mice. Statistical significance was defined using the Mann–Whitney *U*-test (*p < 0.05; **p < 0.005)

To verify the impact of the individual hypoxia pathway proteins, we took advantage of the CD68:cre-PHD2^f/f^ mouse line (henceforth designated P2) [2, 25] and a newly created CD68:cre-HIF1α^f/f^ line (henceforth designated H1). Similar to our findings in P2H1 mice, P2 mice exhibited significantly lower levels of epinephrine in the adrenal glands compared to their WT littermates (Fig. 2A), along with reduced *Pnmt* mRNA and PNMT enzyme activity (Fig. 2B). *Th* and *Dbh* levels remained unaffected in P2 mice (Supplementary Fig. 2). However, compared to WT mice, H1 mice showed no differences in any of hormones measured (Fig. 2C), strongly suggesting that loss of HIF1α alone does not play a significant role in catecholamine production in the adrenal gland. Importantly, these results support our initial observation that PHD2 alters epinephrine synthesis, probably due to HIF2α stabilization.

#### Increased EPO is associated with PNMT-independent reduction in adrenal epinephrine synthesis

**Fig. 3:**
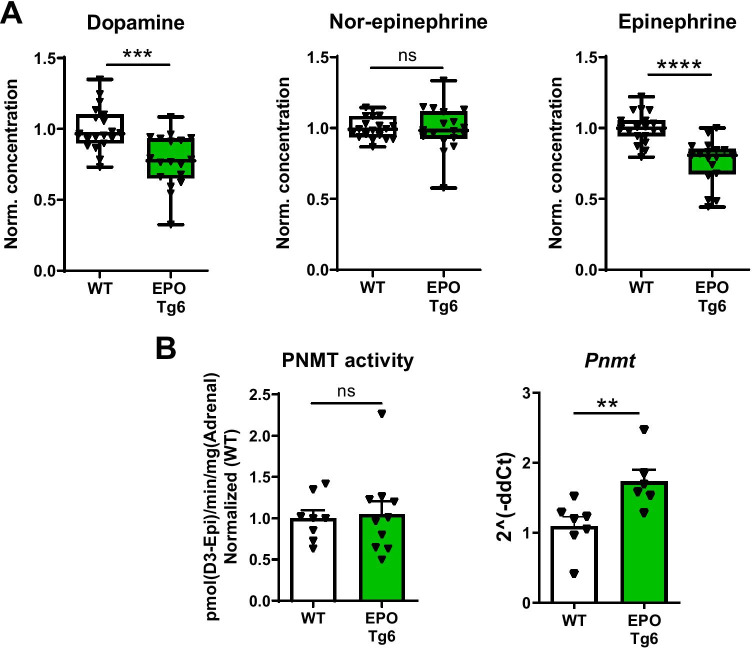
Lower epinephrine in the adrenals of EPO Tg6 mice. **A** Box and whisker plots comparing catecholamine measurements in the adrenals from EPO Tg6 mice and WT littermates (*n* = 19 vs 18 individual adrenal glands). All data are normalized to average measurements in WT mice. The graphs are a representative result of at least 3 independent experiments. **B** PNMT enzyme activity and qPCR-based mRNA expression analysis of *Pnmt* in the adrenals of individual mice (*n* = 8 vs 10). All data are normalized to the average of measurements in WT mice. Statistical significance was defined using the Mann–Whitney *U*-test (*p < 0.05; **p < 0.005)

We have previously shown that both P2 and P2H1 mice, but not H1 mice, exhibit EPO-induced erythrocytosis [25] (Supplementary Fig. 1A); we therefore investigated the potential relationships between reduced epinephrine levels in the adrenal glands and EPO-associated changes. Therefore, we tested the EPO transgenic mouse line (EPO Tg6) [38], which are known to constitutively express high levels of EPO leading to increased RBCs without influencing adrenal hypoxia pathway proteins (Supplementary Fig. 3A-B). Interestingly, similar to P2 and P2H1 mice, epinephrine along with dopamine levels was significantly lower in the adrenal glands of EPO Tg6 mice (Fig. 3A). In contrast to P2H1 and P2 mice, however, no differences in PNMT activity were observed, despite increased *Pnmt* mRNA levels (Fig. 3B). Congruently, mRNA analysis on whole adrenals revealed no changes in *Th* or *Dbh* (Supplementary Fig. 3B). Taken together, these observations suggest that PNMT-independent reduction in adrenal epinephrine levels correlated to high systemic EPO.

#### EPO/erythropoiesis regulates epinephrine release from the adrenal gland

**Fig. 4:**
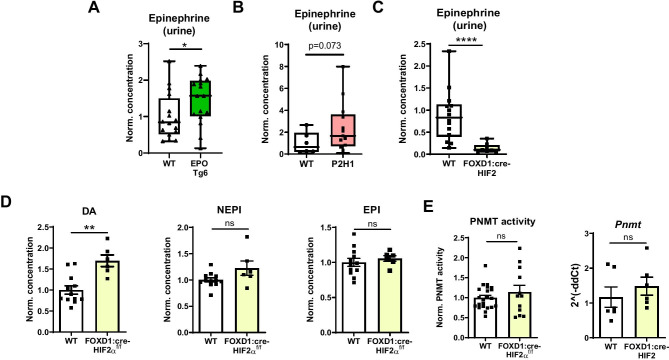
Modulated urinary epinephrine in erythrocytotic and anemic mice. **A** Box and whisker plots comparing urinary epinephrine in EPO Tg6 mice and WT littermates (*n* = 16 vs 16 mice). **B** Box and whisker plots comparing urinary epinephrine in P2H1 mice and WT littermates (*n* = 6 vs 13 mice). **C** Box and whisker plots comparing urinary epinephrine in FOXD1:cre-HIF2α^f/f^ mice and WT littermates (*n* = 14 vs 9 mice). All urine measurements were normalized to urinary creatinine and data was further normalized to average measurements in WT mice. The graphs are a representative result of at least 3 independent experiments. **D** Catecholamine measurements in the adrenals from FOXD1:cre-HIF2α^f/f^ mice compared to WT littermates (*n* = 12 vs 6 individual adrenal glands). Data were further normalized to the average measurements in WT mice. The graphs are a representative result of at least 2 independent experiments. **E** PNMT enzymatic activity and qPCR-based mRNA expression analysis of *Pnmt* measurements in the adrenal of individual mice (*n* = 8 vs 10). All data are normalized to average measurements in WT mice. Statistical significance was defined using the Mann–Whitney *U*-test (*p < 0.05; **p < 0.005)

To characterize the impact of systemic EPO/erythropoiesis on epinephrine in adrenal glands, we looked at potential changes in secretion by measuring epinephrine in urine and found a marked increase in urinary epinephrine in EPO Tg6 mice (Fig. 4A), suggesting either direct or indirect effect of EPO on epinephrine release from the adrenal gland. Similarly, and despite PNMT-dependent inhibition of epinephrine in the adrenal gland (Fig. 1A–B), P2H1 mice showed an almost significant increase in urine epinephrine compared to WT littermates (Fig. 4B).

To further investigate these findings, we used a previously described anemic mouse strain, namely, FOXD1:cre-HIF2α^f/f^ [28]. These mice show low systemic EPO levels and a dramatic reduction in circulatory RBCs (Supplementary Fig. 4A) [28]. In direct contradiction to urinary data of the erythrocytotic EPO Tg6 mice, FOXD1:cre-HIF2α^f/f^ mice showed a > eightfold reduction in urinary epinephrine compared to WT littermates (Fig. 4C). Interestingly, the adrenal glands of these anemic mice showed a significant increase in dopamine, while levels of all other catecholamines remained unchanged (Fig. 4D). Levels of neither PNMT (Fig. 4E) nor of any of the other corresponding enzymes were altered (Supplementary Fig. 4B). Taken together, at this point, our data strongly point towards EPO-mediated exocytosis of epinephrine and which is independent of the PHD2/HIF2/PNMT axis regulating the synthesis of epinephrine in the chromaffin cells.

#### EPO-induced hypoglycemia activates epinephrine release

**Fig. 5:**
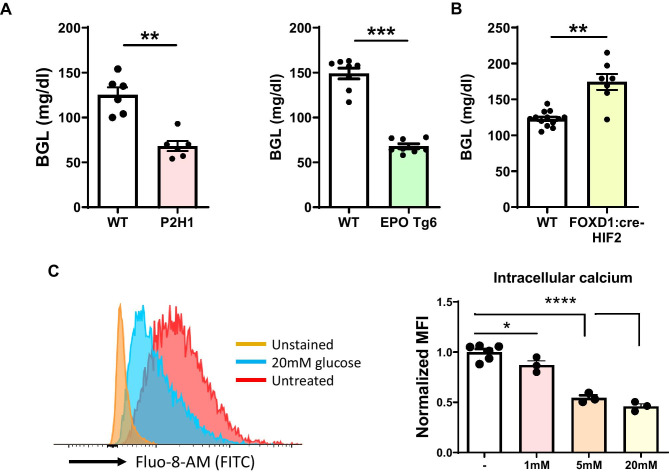
EPO-mediated regulation of blood glucose levels in erythrocytotic and anemic mice. **A** Blood glucose level (BGL) measurements in P2H1 and EPO Tg6 mice compared to WT littermates (*n* = 6–8 mice). **B** BGL in FOXD1:cre-HIF2α^f/f^ mice compared to WT littermates (*n* = 6–13 mice). **C** Representative histograms showing fluorescence of the Fluo-8-Am dye for intracellular calcium measurement in untreated MPC cells compared to stimulation with glucose. Normalized MFI depicting intracellular calcium measurement in MPC cells that were treated with glucose in a dose-dependent manner. Each dot represents an individual well. The data is representative of 2 individual experiments. Statistical significance was defined using the Mann–Whitney *U*-test or unpaired *t*-test (*p < 0.05; **p < 0.005)

Epinephrine release from chromaffin cells requires their exocytosis and this process is stimulated by hypoglycemia while inhibited by hyperglycemia [17, 20]. Recently, it has been shown that hyperactive erythropoiesis, as seen in EPO Tg6 mice, increases systemic glucose consumption and consequent hypoglycemia, which is most probably attributable to a greater number of circulating RBCs [22]. Therefore, we sought to understand if and how EPO-associated hypoglycemia can affect adrenal epinephrine release. We first measured blood glucose levels (BGLs) in all mouse lines and found that both erythrocytotic P2H1 and EPO Tg6 mice display significant hypoglycemia (Fig. 5A), while conversely, the anemic FOXD1:cre-HIF2α^f/f^ mice were dramatically hyperglycemic (Fig. 5B). Next, we used a mouse pheochromocytoma cell line (MPC) to study how changes in blood glucose levels could affect cellular exocytosis. As these cells do not produce measurable amounts of epinephrine, we measured the uptake of calcium upon glucose stimulation, directly correlating with the degree of epinephrine exocytosis [39, 40]. Therefore, we exposed MPCs to increasing concentrations of glucose and observed a significant inhibition of calcium uptake, indicating reduced exocytosis (Fig. 5C). In contrast, direct exposure of MPCs to high EPO did not lead to changes in intracellular calcium (Supplementary Fig. 5A and B), clearly suggesting that EPO would not directly influence epinephrine exocytosis; rather, it is systemic hypoglycemia secondary to EPO-induced erythrocytosis in mice that enhances adrenal epinephrine release. Conversely, anemia induces hyperglycemia resulting in the inhibition of epinephrine release.

### Discussion

**Fig. 6:**
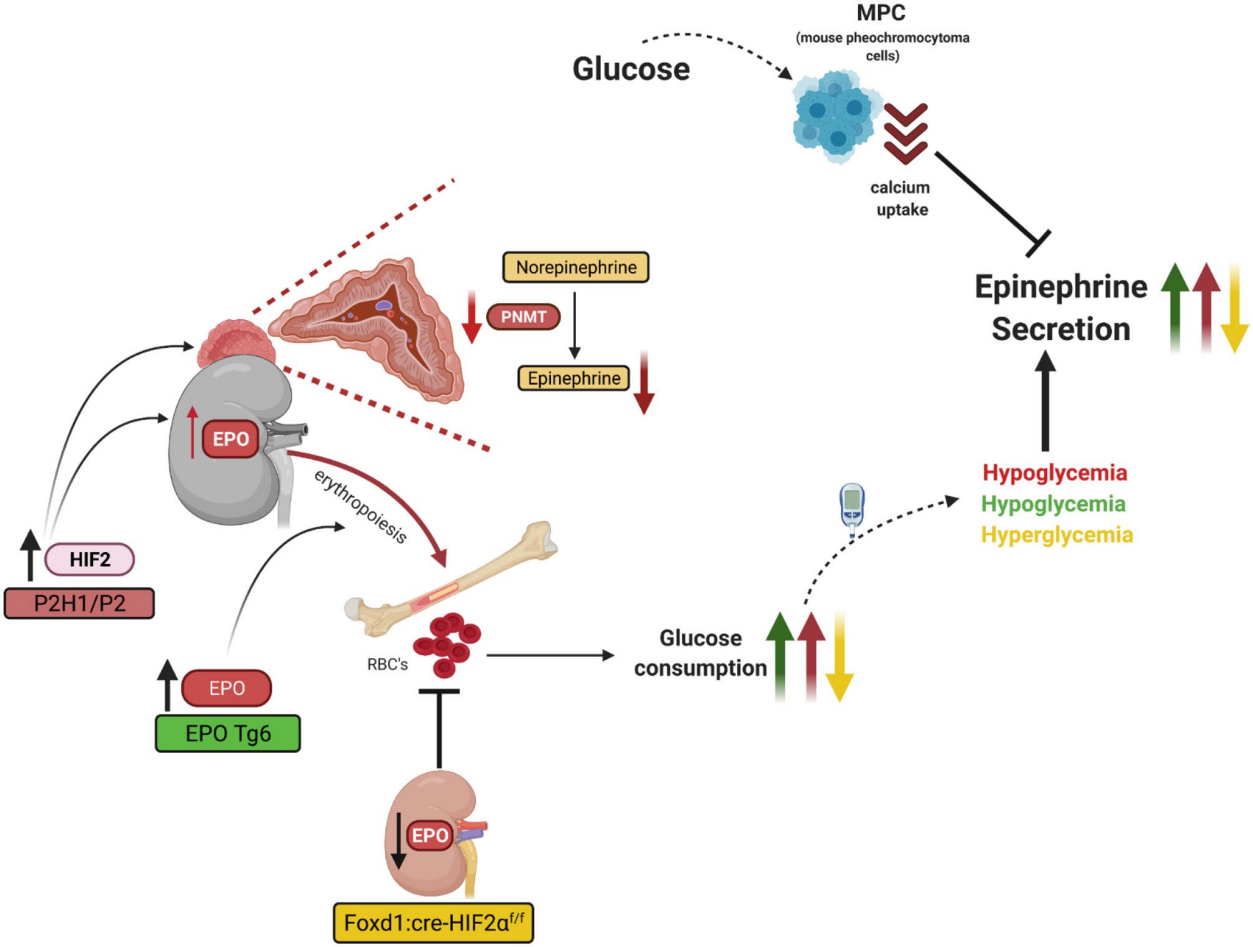
Graphical overview of the PHD2-HIF2α axis involved in synthesis and secretion of epinephrine from the mouse adrenal gland

Here, we show that decreased PNMT expression in the PHD2-deficient adrenal medulla reduces epinephrine synthesis, but systemic effects in these PHD2-deficient mice, such as enhanced EPO production, consequent RBC excess, and hypoglycemia, lead to enhanced secretion of this hormone from the adrenal gland (Fig. 6). These results indicate uncoupling between synthesis and secretion of epinephrine upon PHD2 loss leading to reduced size of adrenal epinephrine stores. Mechanistically, we show that excessive epinephrine secretion is related to EPO-induced systemic hypoglycemia, rather than a direct cell-level effect of EPO per se.

Multiple hypoxia pathway components have been suggested to define the functionality of the adrenal medulla. In the physiological sympathoadrenal setting, pVHL has been shown to be essential during development and it is required by the peripheral oxygen-sensing system to ensure survival under hypoxic conditions [41]. Loss-of-function mutations in the *VHL* gene or gain-of-function mutations in *HIF2α* have been associated with the development of pheochromocytoma and paraganglioma [42]. Previously, it was suggested that HIF2α is essential for catecholamine homeostasis during embryonic development [43]. However, this HIF subunit is not essential for the development and functionality of the adrenal medulla, as mice deficient for HIF2α in TH-positive medullary cells display no functional abnormalities [44]. In contrast, a novel mouse line containing a HIF2α gain-of-function mutation has been reported to display reduced adrenal PNMT and urinary metanephrine/normethanephrine ratios, although no data on potential modulation of catecholamines in the adrenal gland were provided [14]. Our data on HIF2α-induced reduction in PNMT activity concur with these observations. Likewise, several in vitro studies have described the effects of HIF2α on the various enzymes involved in the process of catecholamine production in chromaffin-related cell lines [7–9, 45, 46]. Our findings that HIF2α induction in medullar cells results in the repression of *Pnmt* mRNA and its activity appear counterintuitive but might be the result of an indirect effect driven by transcriptional repressors. In line with this, transcriptional regulation of adrenal steroidogenesis has already been connected to miRNAs, some of which might be directly regulated by hypoxia/HIF [47, 48]. Therefore, more research is required to elucidate the direct or indirect impact of HIF2α on the production of epinephrine. The role of HIF1α in the adrenal medulla remains much less explored, despite results suggesting indirect activation of the *Pnmt* promotor by HIF1α via the transcription factors Egr-1 and Sp1 [49]. Therefore, further insight into how PHDs/HIFs regulate epinephrine production and release from the adrenal medulla in an in vivo setting is essential to further implement this knowledge in the promising field of HIF stabilizers (HIF-PHD inhibitors) in the treatment of anemia. However, it must be noted here that concerns have been recently raised regarding the chronic use of such HIF stabilizers [3, 50–53].

The advantage of our approach lies in the use of multiple transgenic mice lines to define the individual roles of PHD2 and HIF1α in epinephrine physiology, which was complimented by studies in mice that are conditionally deficient for both PHD2 and HIF1α in cells of neural origin, i.e., REPCs [25, 54]. Observations from all these mouse lines strongly suggest targeting of chromaffin cells in the adrenal medulla. We have previously reported HIF2α stabilization in P2H1 mice and demonstrated that the excessive erythrocytosis phenotype seen in these P2H1 mice is attributable to HIF2α stabilization in the absence of the protective effects of HIF1α [25]. Similarly, we show that loss of PHD2 alone, but not of HIF1 alone, resembled the P2H1 phenotype with respect to changes in epinephrine synthesis and secretion, indicating that this phenotype too is enhanced through stabilization of HIF2α and that HIF1α might not have a significant role. Further, although we found a comparable decrease in epinephrine levels in P2H1 and P2 adrenal glands and a corresponding reduction in adrenal PNMT activity, urinary excretion of epinephrine was higher in P2H1 mice, suggesting that epinephrine was preferentially released from the adrenal glands. As P2H1 mice also display excessive erythrocytosis that is associated with very high levels of EPO, we hypothesized that this excessive EPO was responsible for the observed preferential release of epinephrine. Therefore, we used erythrocytotic EPO Tg6 mice to understand the effects of only the EPO/polycythemia phenotype on epinephrine physiology without interference from the HIF2-PNMT pathway [38], and show that excessive EPO indeed increases urinary epinephrine excretion, thereby supporting our hypothesis.

Under basal conditions, exocytosis from the chromaffin cells is a strictly controlled process that largely depends on calcium uptake [55–58]. However, it can be further enhanced by additional stimuli, including hypoglycemia, hypotension, hypoxemia, and emotional distress [5, 16, 58, 59]. Interestingly, it has been recently reported that EPO Tg6 mice are strongly hypoglycemic and that low BGL correlates with the degree of erythropoiesis [22, 60]. Moreover, it was postulated almost a century ago that hypoglycemia can per se stimulate epinephrine release from the adrenal medulla [61]. The results of our in vitro experiments in MPCs lend support to this hypothesis as we show that calcium uptake reduces as glucose concentration increases, and that direct EPO exposure does not produce such an effect. Thus, based on our observations, we suggest, for the first time, that EPO-induced RBC excess, and consequent hypoglycemia, leads to enhanced exocytosis of epinephrine that is mediated by enhanced Ca^2+^ uptake in chromaffin cells. Further, higher *Pnmt* mRNA and reduced dopamine levels in EPO Tg6 adrenal glands are most likely the consequence of such enhanced exocytosis. These conclusions are borne out by the contrasting observations in the anemic FOXD1:cre-HIF2α^f/f^ mice [28], which show dramatically low levels of epinephrine in urine that were linked to hyperglycemia, even though adrenal epinephrine and PNMT levels remained unchanged.

In summary, we show that PHD2-mediated HIF2α stabilization in the offspring of neural crest cells has divergent local and systemic effects in vivo, i.e., while epinephrine synthesis is diminished, its urinary excretion is enhanced. Importantly, in these mice, the adrenal synthesis of epinephrine and its urinary secretion appear to be clearly uncoupled as excessive urinary epinephrine secretion appears to be due to systemic EPO-induced effects via the PHD2-HIF2-EPO axis, which include excessive erythrocytosis and consequent increase in glucose consumption by these RBCs. Mechanistically, we propose that the EPO-induced RBC excess and consequent hypoglycemia lead to enhanced exocytosis of epinephrine, irrespective of the primary reduction in adrenal epinephrine synthesis. These findings have potential clinical significance in view of broad spectrum HIF-PHD inhibitors currently under phase III trials.

## Supplementary Information

Below is the link to the electronic supplementary material.

Supplementary file1 (DOCX 17 KB)

Supplementary file2 (PPTX 1419 KB)

## Data Availability

All data and material are available upon reasonable request to ben.wielockx@tu-dresden.de.
