# Supplementary material for: HIF2α regulates the synthesis and release of epinephrine in the adrenal medulla
Source: J Mol Med (Berl). 2021 Sep 4;99(11):1655–66. doi: 10.1007/s00109-021-02121-y (PMC8542008; doi:10.1007/s00109-021-02121-y)
Supplement: Supplementary file 2 — Supplementary file2 (PPTX 1419 KB) [file 109_2021_2121_MOESM2_ESM.pptx]

## Slide 1
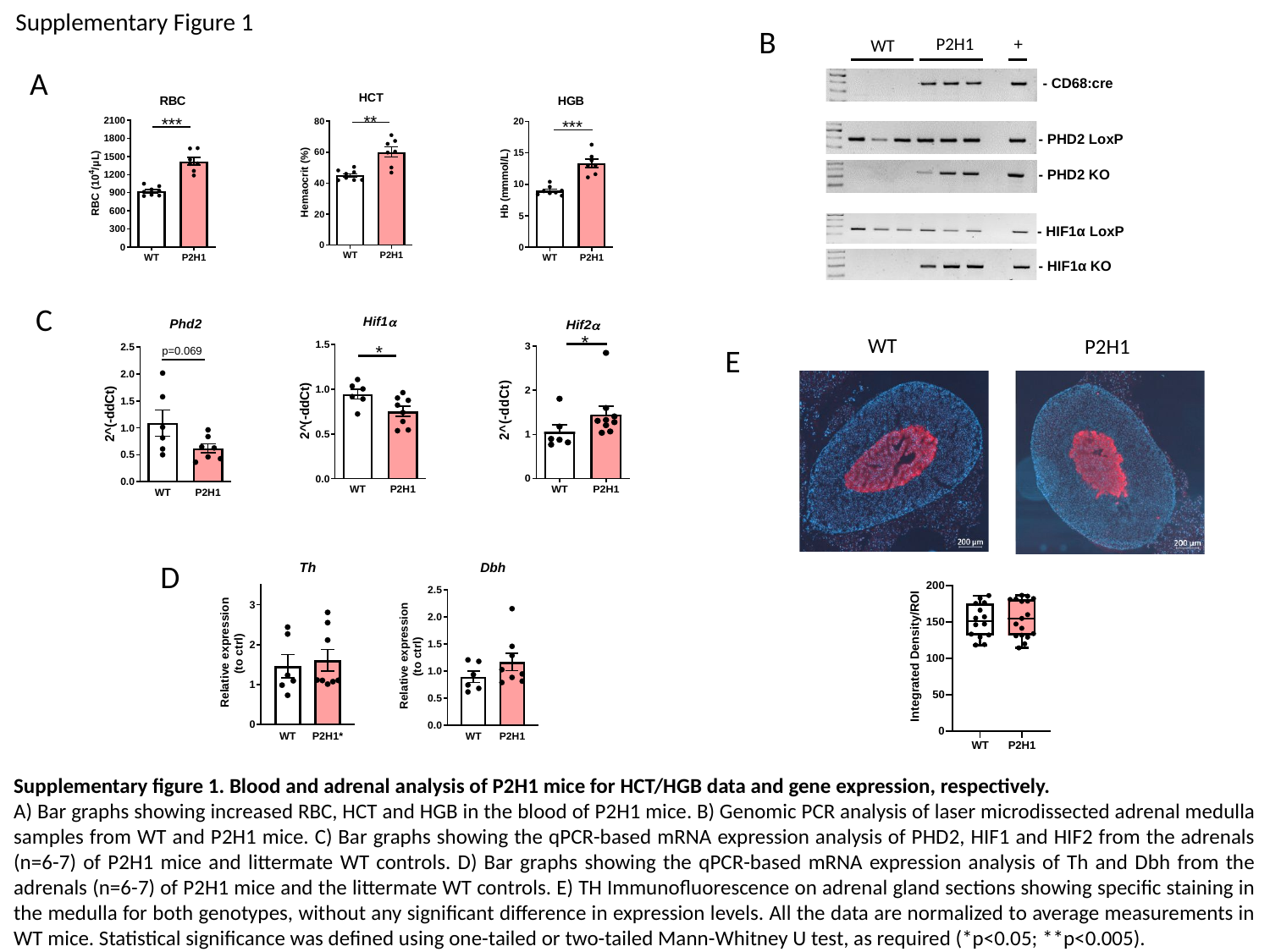

Supplementary Figure 1
B
 P2H1 +
WT
A
- CD68:cre
- PHD2 LoxP
- PHD2 KO
- HIF1α LoxP
- HIF1α KO
C
WT
P2H1
E
D
Supplementary figure 1. Blood and adrenal analysis of P2H1 mice for HCT/HGB data and gene expression, respectively.
A) Bar graphs showing increased RBC, HCT and HGB in the blood of P2H1 mice. B) Genomic PCR analysis of laser microdissected adrenal medulla samples from WT and P2H1 mice. C) Bar graphs showing the qPCR-based mRNA expression analysis of PHD2, HIF1 and HIF2 from the adrenals (n=6-7) of P2H1 mice and littermate WT controls. D) Bar graphs showing the qPCR-based mRNA expression analysis of Th and Dbh from the adrenals (n=6-7) of P2H1 mice and the littermate WT controls. E) TH Immunofluorescence on adrenal gland sections showing specific staining in the medulla for both genotypes, without any significant difference in expression levels. All the data are normalized to average measurements in WT mice. Statistical significance was defined using one-tailed or two-tailed Mann-Whitney U test, as required (*p<0.05; **p<0.005).

## Slide 2
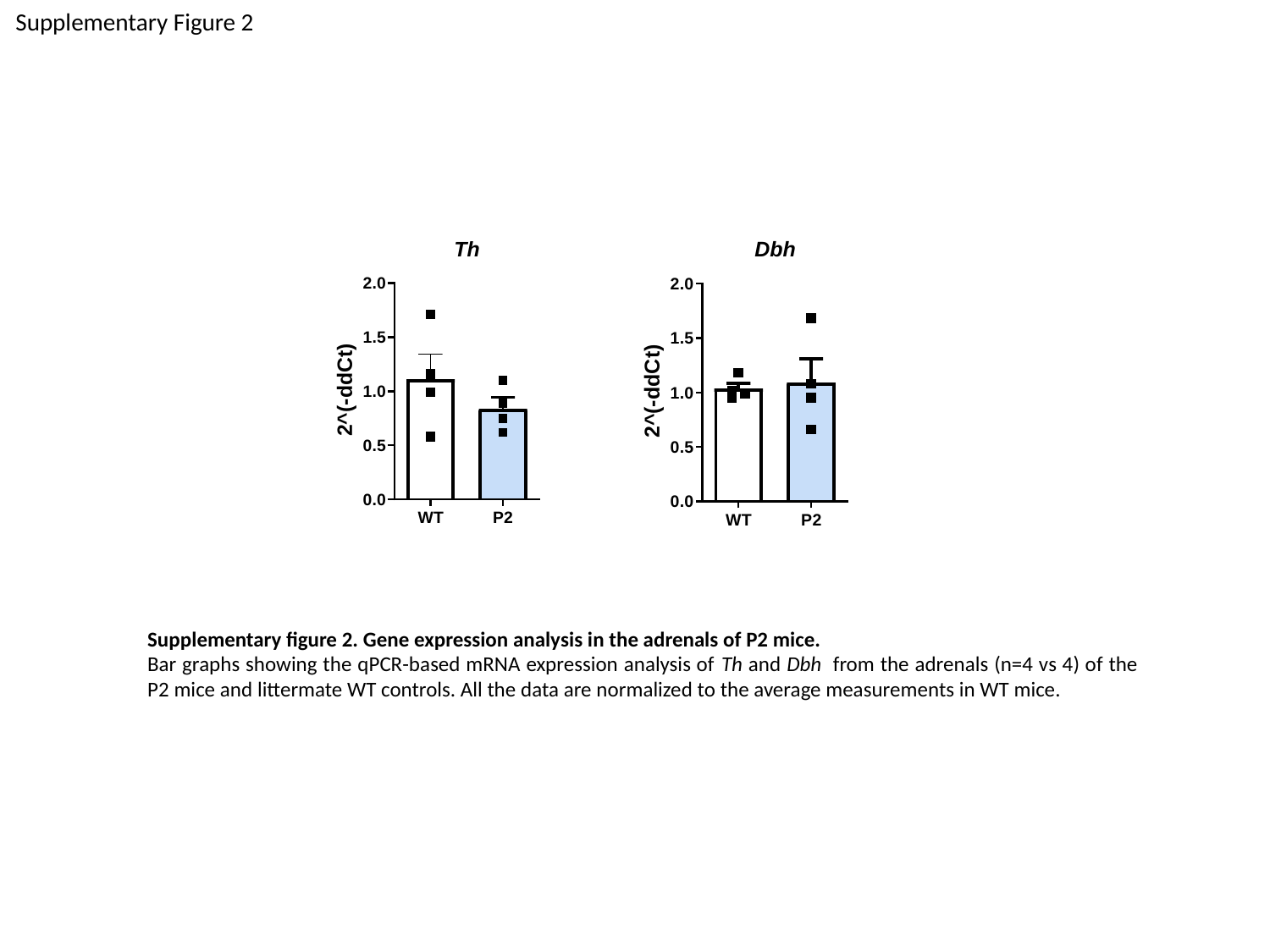

Supplementary Figure 2
Supplementary figure 2. Gene expression analysis in the adrenals of P2 mice.
Bar graphs showing the qPCR-based mRNA expression analysis of Th and Dbh from the adrenals (n=4 vs 4) of the P2 mice and littermate WT controls. All the data are normalized to the average measurements in WT mice.

## Slide 3
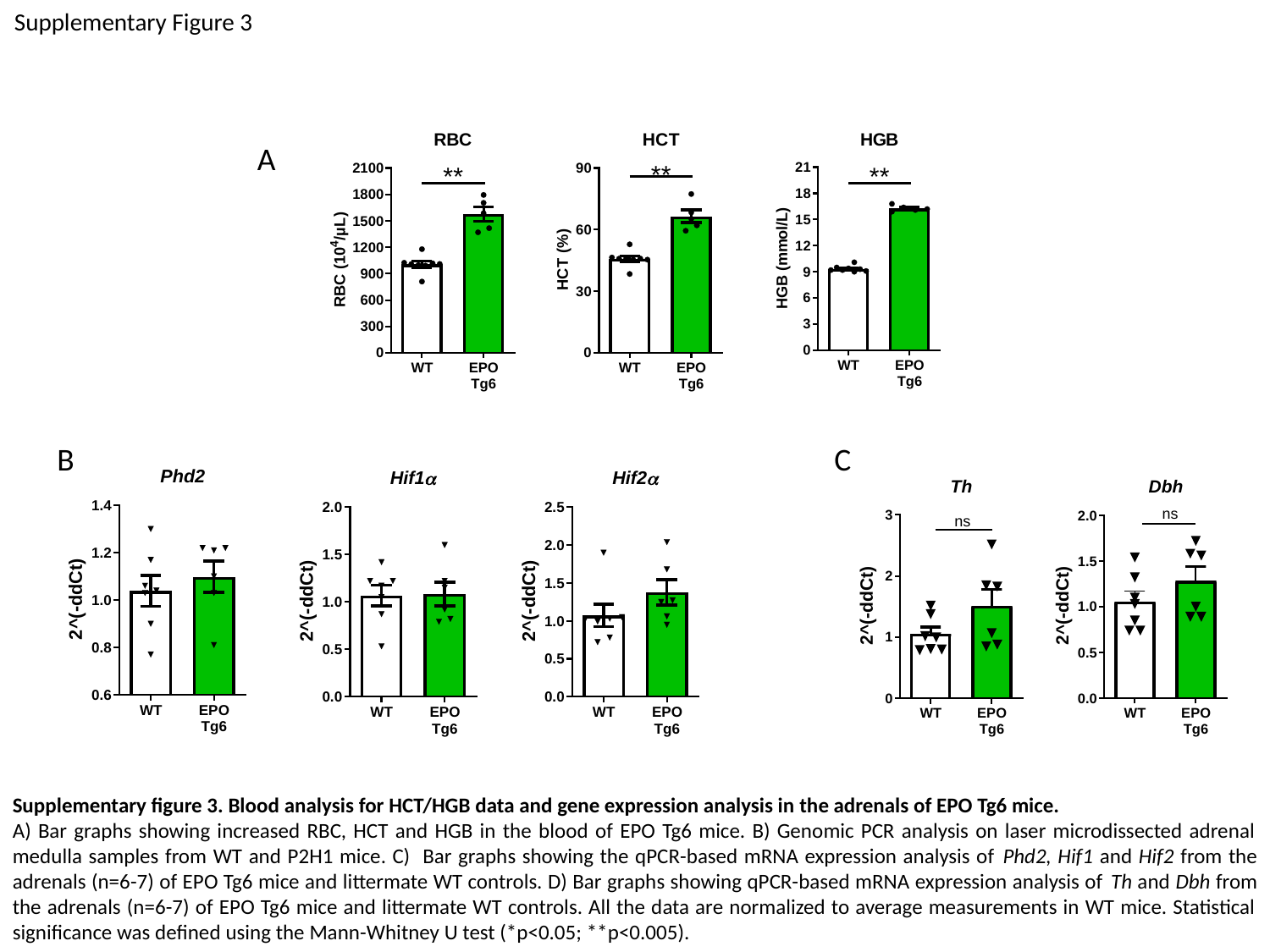

Supplementary Figure 3
A
B
C
Supplementary figure 3. Blood analysis for HCT/HGB data and gene expression analysis in the adrenals of EPO Tg6 mice.
A) Bar graphs showing increased RBC, HCT and HGB in the blood of EPO Tg6 mice. B) Genomic PCR analysis on laser microdissected adrenal medulla samples from WT and P2H1 mice. C) Bar graphs showing the qPCR-based mRNA expression analysis of Phd2, Hif1 and Hif2 from the adrenals (n=6-7) of EPO Tg6 mice and littermate WT controls. D) Bar graphs showing qPCR-based mRNA expression analysis of Th and Dbh from the adrenals (n=6-7) of EPO Tg6 mice and littermate WT controls. All the data are normalized to average measurements in WT mice. Statistical significance was defined using the Mann-Whitney U test (*p<0.05; **p<0.005).

## Slide 4
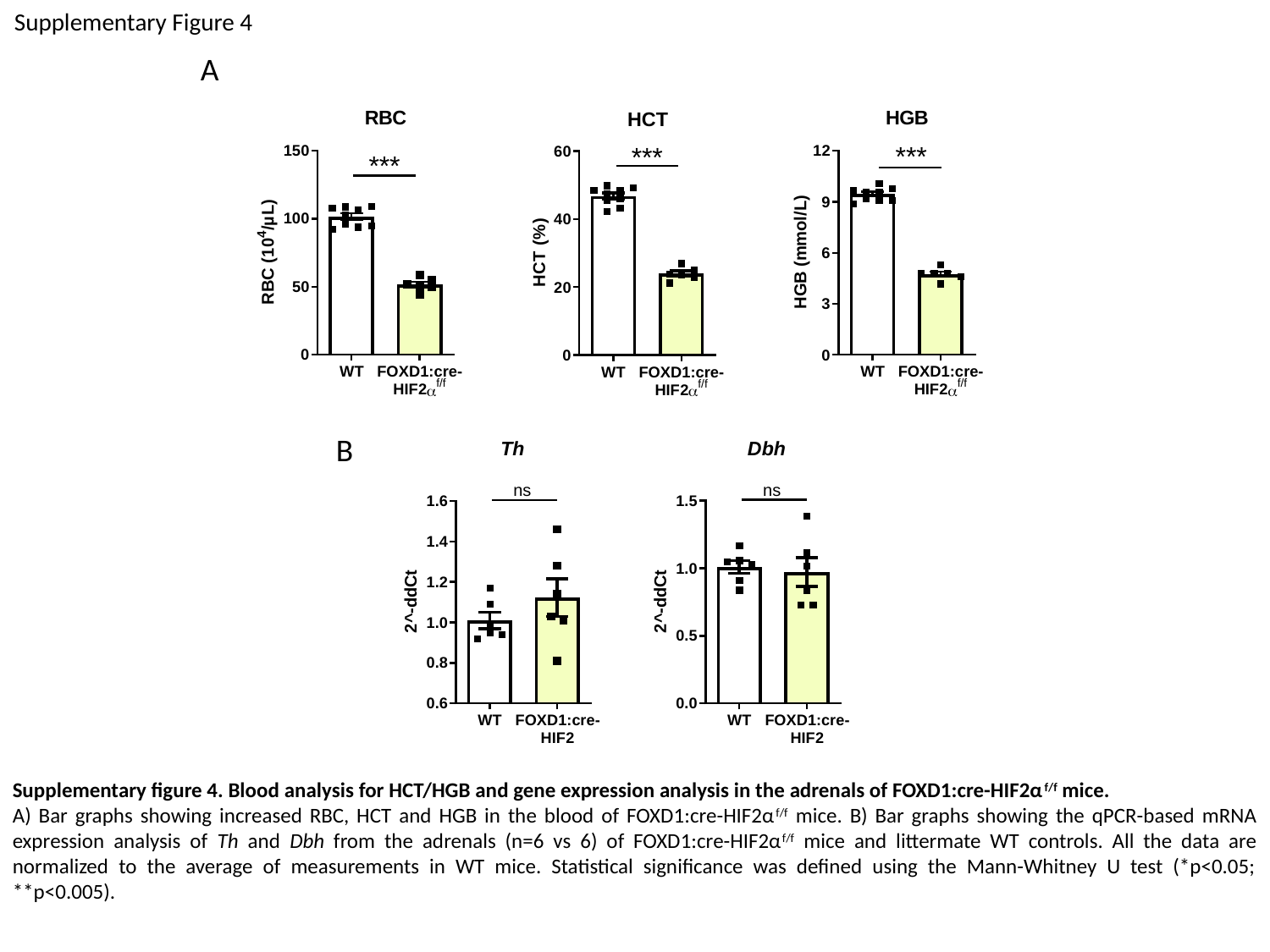

Supplementary Figure 4
A
B
Supplementary figure 4. Blood analysis for HCT/HGB and gene expression analysis in the adrenals of FOXD1:cre-HIF2αf/f mice.
A) Bar graphs showing increased RBC, HCT and HGB in the blood of FOXD1:cre-HIF2αf/f mice. B) Bar graphs showing the qPCR-based mRNA expression analysis of Th and Dbh from the adrenals (n=6 vs 6) of FOXD1:cre-HIF2αf/f mice and littermate WT controls. All the data are normalized to the average of measurements in WT mice. Statistical significance was defined using the Mann-Whitney U test (*p<0.05; **p<0.005).

## Slide 5
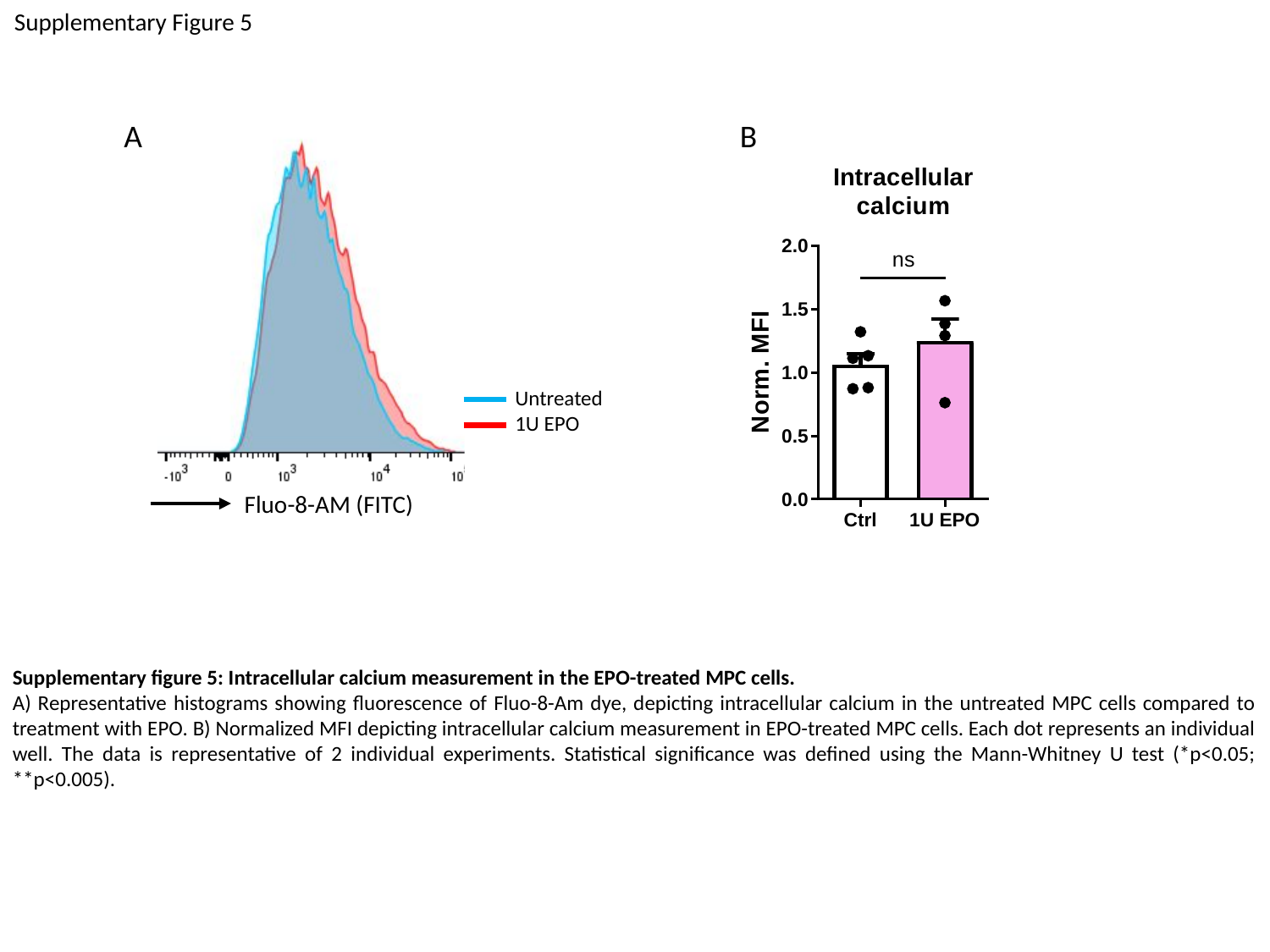

Supplementary Figure 5
A
B
Untreated
1U EPO
Fluo-8-AM (FITC)
Supplementary figure 5: Intracellular calcium measurement in the EPO-treated MPC cells.
A) Representative histograms showing fluorescence of Fluo-8-Am dye, depicting intracellular calcium in the untreated MPC cells compared to treatment with EPO. B) Normalized MFI depicting intracellular calcium measurement in EPO-treated MPC cells. Each dot represents an individual well. The data is representative of 2 individual experiments. Statistical significance was defined using the Mann-Whitney U test (*p<0.05; **p<0.005).
